# Pregnancy-Related Complications During the COVID-19 Pandemic in Iran

**DOI:** 10.34172/aim.2024.05

**Published:** 2024-01-01

**Authors:** Maryam Gharacheh, Narjes Khalili, Mohammad Ebrahimi Kalan, Mohammad Heidarzadeh, Fahimeh Ranjbar

**Affiliations:** ^1^Nursing and Midwifery Care Research Center, Health Management Research Institute, Iran University of Medical Sciences, Tehran, Iran; ^2^Preventive Medicine and Public Health Research Center, Psychosocial Health Research Institute, Department of Community and Family Medicine, School of Medicine, Iran University of Medical Sciences, Tehran, Iran; ^3^School of Health Professions, Eastern Virginia Medical School, Norfolk, VA, USA; ^4^Department of Pediatrics, School of Medicine, Children Medical Research and Training Hospital, Tabriz University of Medical Sciences, Tabriz, Iran

**Keywords:** COVID-19 pandemic, Maternal health, Pregnancy complications, SARS-CoV-2

## Abstract

**Background:**

The COVID-19 pandemic has profoundly affected healthcare systems worldwide, with significant collateral damage to vulnerable populations, including the perinatal population. This study sought to compare pregnancy-related complications before and during the COVID-19 pandemic in Iran.

**Methods:**

This retrospective data analysis was performed from February 20 to August 20, 2019 (prior to the onset of the COVID-19 pandemic) and from February 20 to August 20, 2020 (during the pandemic), encompassing the initial wave of the pandemic and the subsequent lockdown. To collect data, we utilized the medical records of 168,358 women obtained from the Iranian Maternal and Neonatal Network, which is a comprehensive electronic health record database management system specifically designed to store information pertaining to maternal and neonatal health.

**Results:**

A total of 168,358 medical records were analyzed, with 87388 (51.9%) and 80970 (48.1%) before and during the pandemic, respectively. The occurrence of pregnancy complications was found to be significantly more frequent during the pandemic compared to the pre-pandemic period. Notably, there was a higher likelihood of experiencing preeclampsia (odds ratio [OR]=1.14, 95% confidence interval [CI]: 1.07‒1.22, *P*=0.0001) and gestational diabetes (OR=1.14, 95% CI: 1.09‒1.19, *P*=0.0001) during the pandemic. Furthermore, cesarean section (CS) became more prevalent during the pandemic in comparison to vaginal delivery (OR=1.19, 95% CI: 1.17‒-1.22, *P*=0.0001).

**Conclusion:**

Our findings demonstrated a significant association between the COVID-19 pandemic and an escalation in adverse pregnancy outcomes, notably preeclampsia, gestational diabetes, and CS deliveries. However, further research is warranted to gain a richer understanding of the intricate interplay between the COVID-19 pandemic and pregnancy complications. This is particularly crucial in light of the evolving landscape of new coronavirus variants.

## Introduction

 The COVID-19 pandemic is still wreaking havoc on families, the economy, and the healthcare system, infecting over 405 million people and killing more than 5.7 million globally.^1^ This pandemic has extensively changed most people’s way of life and disrupted general and specialist healthcare systems. The healthcare disruptions induced by the COVID-19 pandemic have resulted in significant adverse consequences, particularly for vulnerable populations, such as the perinatal population.^2^ Pregnancy constitutes a risk factor for heightened morbidity and mortality during the outbreak of numerous illnesses, such as acute respiratory infections.^3^ The detrimental effect of the COVID-19 pandemic on maternal and perinatal health extends beyond the direct mortality and morbidity caused by the SARS-CoV-2 infection. The implementation of nationwide lockdowns, healthcare service disruptions, and fear of attending healthcare centers might have influenced the health of pregnant women and their infants.^4^

 Recent evidence suggests that rates of pregnancy-related complications might have changed considerably during the pandemic.^5^ Pregnancy complications increase a woman’s risk for maternal mortality.^6^ Globally, a worrisome estimation shows that COVID-19 led to about a 39% rise in maternal death and a 45% increase in child death per month across 118 low- and middle-income countries (LMICs).^7,8^ According to a systematic review and meta-analysis, the COVID-19 pandemic has had a detrimental effect on global maternal and fetal outcomes, with a rise in ruptured ectopic pregnancies, maternal deaths, and stillbirths in LMICs.^4^ Furthermore, a recent US study has demonstrated a significant association between the COVID-19 pandemic and an elevated risk of gestational diabetes, gestational hypertension, preeclampsia, and compromised fetal growth.^5^ This adverse trend could be attributed to factors such as diminished health-care-seeking behavior, reduced availability of comprehensive maternity services, and limited access to antenatal care, including interventions for managing pre-eclampsia and providing supplementation guidance.^7,9^ It is noteworthy that even a moderate decline of 10% in the coverage of maternal and neonatal healthcare could potentially lead to a further 28 000 maternal and 168 000 neonatal deaths worldwide.^10^

 The effect of the pandemic is likely to be amplified during the preparedness stages in LMICs with limited resources. Even prior to the beginning of the COVID-19 pandemic, these countries faced challenges such as inadequate availability, limited accessibility, and high costs associated with acquiring quality and timely maternal and child health services.^11^ In Iran, multifaceted measures were implemented to control the spread of the SARS-CoV‐2 infection, commencing on February 19, 2020. These measures included the cancelation of public events, the closure of educational institutions, holy shrines, and shopping centers, as well as the prohibition of festival celebrations. Additionally, dedicated outposts were set up at city entrances for detecting coronavirus cases, and a hotline was established to facilitate the identification of COVID-19 cases and provide relevant health information. Despite earnest efforts to curtail the spread of COVID-19, Iranian authorities encountered numerous challenges while combating the pandemic. These included shortages of essential personal protective equipment such as masks and disinfectants, insufficient hospital beds and equipment, and a shortage of healthcare providers.^12^ It is plausible that these factors exerted an impact on pregnancy outcomes.

 Considering that this pandemic is still wreaking havoc on healthcare systems worldwide and the initial evidence has been inconsistent, robust data on the effect of the COVID-19 pandemic on pregnancy complications is necessary.^5^ In addition, changes in the health policies of the affected countries, frequently evolving clinical management guidelines and uncertainty around the reliability of research findings, make it crucial to consistently monitor health complications before and after pregnancy during this pandemic and how they differ from the pre-pandemic period.^13^ Therefore, it is critical for the scientific community to generate evidence-based clinical and epidemiological links between COVID-19 and pregnancy outcomes.^14^ The invaluable insights derived from research studies play a pivotal role in the development of clinical recommendations and the formulation of context-specific public health guidance.^15^ Hence, this study aimed to compare pregnancy-related complications before and during the COVID-19 pandemic in Iran.

## Materials and Methods

 Using retrospective analysis, we examined the electronic medical records of women who were admitted to the maternity department of hospitals in Tehran prior to the onset of the COVID-19 pandemic (February 20 to August 20, 2019) and during the pandemic (February 20 to August 20, 2020), encompassing the initial wave of the pandemic and the subsequently implemented lockdown measures.

 To collect data, we utilized the Iranian Maternal and Neonatal Network, which serves as a comprehensive health record database management system specifically designed to gather and manage maternal and neonatal information across the entire country. Within this comprehensive system, we examined various aspects, including pregnancy complications (e.g., gestational diabetes, preeclampsia, anemia, preterm birth, and cesarean delivery) and maternal characteristics (e.g., maternal age, education level, gestational age, parity, gravidity, abortion, and living children).

###  Definitions

*Preeclampsia* is referred to as the occurrence of elevated blood pressure and proteinuria, or elevated blood pressure and end-organ dysfunction with or without proteinuria, after 20 weeks of gestation in a woman who was previously normotensive.^16^

*Gestational diabetes* is characterized by elevated blood glucose levels, typically detected after 24 weeks of gestation.^17^ The diagnosis was confirmed when one or more of the following criteria were met:

 Fasting plasma glucose (FPG) ≥ 126 mg/dL or random plasma glucose ≥ 200, with confirmation required by FPG or glycated hemoglobin.

*Preterm birth *is referred to as the delivery of an infant before 37 weeks of completed gestation.^18^

*Anemia* was considered a hemoglobin level below 11.0 g/dL during the first trimester and below either 10.5 or 11.0 g/dL during the second or third trimester.^19^

*Miscarriage* is referred to as the event of pregnancy loss occurring within the first 22 weeks of gestation.

###  Statistical Analysis

 Sample frequencies and percentages, as well as sample means and standard deviations, were reported for categorical and continuous variables, respectively. Univariate logistic regression analysis was conducted for each pregnancy complication, considering pre- vs. intra-pandemic as an outcome. The crude odds ratio (OR) and corresponding 95% confidence interval (CI) were reported. Next, four multivariable logistic regression models were performed for each pregnancy complication separately to obtain differences between intra- vs. pre-pandemic pregnancy complications, accounting for all covariates, including preterm birth, low birth weight, abortion, previous hypertension, previous diabetes, Apgar score, birth order, maternal age, gestational age, birth weight, gravidity, and parity. The confounders were selected based on subject matter knowledge, the availability of indicators, and a literature review. The adjusted OR (aOR) and corresponding 95% CI were reported. All analyses were performed using SAS 9.3, with the statistical significance level being set at α = 0.05. The Box-Tidwell test was performed to assess the linearity assumption for quantitative predictors in our logistic regression models. This test involves regressing the log odds of the outcome variable against a transformed version of the predictor. If the *P* value from this test is non-significant, it indicates that the linearity assumption is met. The only variable that violated the linearity assumption was gestational age. Therefore, gestational age was transformed using the natural logarithm, and the adjusted aORs remained consistent with the untransformed model, reaffirming the suitability of the log transformation.

## Results

 In this study, a comprehensive analysis was conducted on a total of 168,358 medical records. Of these, 87 388 (51.9%) were collected prior to the onset of the COVID-19 pandemic, while the remaining 80 970 (48.1%) were obtained during the pandemic period. The mean age of the mothers included in the study was 30.45 ± 5.8 years. The majority of these mothers had obtained a secondary school level of education. Additionally, the mean ± SD gestational age was found to be 38.07 ± 2.0 weeks among the participants. Furthermore, neonates enrolled in the study had an average birth weight of 3144.3 ± 540.9 g. There was a significant difference in the numbers of pregnancies, deliveries, abortions, and living children (*P*= 0.001) between the pre-pandemic and pandemic periods. As shown in univariate analysis (Table 1), the occurrence of pregnancy complications was more likely to happen during the pandemic period compared to the pre-pandemic period, including preeclampsia (OR = 1.14, 95% CI: 1.07‒1.22, *P* = 0.0001) and gestational diabetes (OR = 1.14, 95% CI: 1.09‒1.19, *P* = 0.0001). Compared to the pre-pandemic period, cesarean section (SC) was more common than normal delivery (OR = 1.19, 95% CI: 1.17‒1.22, *P*= 0.0001) during the pandemic. The number of vaginal deliveries with intervention was lower in the pandemic period in comparison to the pre-pandemic period (15.0% vs. 16.7%, *P* = 0.0001). There was no significant difference in delivery complications (e.g., third-/fourth-degree perineal laceration, blood transfusion, and shoulder dystocia) between pre- and pandemic periods (.3% vs..2%, *P* = 0.45). Multivariable models yielded similar results even after controlling for potential confounders (Table 2). The analysis showed that there was no statistically significant difference in anemia between the two time points, with a *P* value of 0.054. It is crucial to note that this *P*-value, although slightly above the conventional significance threshold of 0.05, does not provide strong evidence against the null hypothesis of no difference.

**Table 1:** Study Sample Characteristics (N = 168 358)

**Characteristics**	**Number**	**%**
Preterm birth, yes	16128	9.6
Abortion, ≥ 1	37920	22.5
Previous hypertension, yes	2801	1.7
Previous diabetes, yes	784	0.5
Preeclampsia, yes	3460	2.1
Gestational diabetes, yes	9283	5.5
Anemia, yes	64	.04
Mode of delivery		
NVD	50002	29.7
CS	118356	70.3
Low birth weight, yes	14829	8.8
Apgar score		
< 7	2358	1.4
7-10	166000	98.6
Birth order		
1^st^	165080	98.0
2^nd^ or more	3278	2.0
	**Mean**	**SD**
Maternal age (y)	30.45	5.8
Gestational age (wk)	38.07	2.0
Birth weight (g)	3144.3	540.9
	**Median**	**IQR**
Gravidity	2	2
Parity	1	1

*Note*. NVD: Normal vaginal delivery; CS: Cesarean section; SD: Standard deviation; IQR: Interquartile range.

**Table 2:** Pregnancy Complications Pre- and Intra-pandemic Periods

**Pregnancy Complications**	**Intra- vs. Pre-pandemic Period**	
	**Crude OR (95% CI), ** ***P *** **Value**	Adjusted OR* (95% CI), ***P *** **Value**
Preeclampsia		
No	Reference	Reference
Yes	1.14 (1.07–1.22), 0.0001	1.14 (1.07-1.22), 0.0002
Gestational diabetes		
No	Reference	Reference
Yes	1.14 (1.09–1.19), < .0001	1.12 (1.09-1.17), < .0001
Anemia		
No	Reference	Reference
Yes	0.61 (0.36–1.00), 0.0542	0.61 (0.36-1.06), 0.0547
Mode of delivery		
Normal delivery	Reference	Reference
Cesarean section	1.19 (1.17–1.22), < .0001	1.19 (1.16-1.21), < .0001

*Note*. OR: Odds ratio; CI: Confidence interval. ^*^A multivariable logistic regression model was performed for each pregnancy complication separately to obtain differences between intra- vs. pre-pandemic pregnancy complications accounting for preterm birth, low birth weight, abortion, previous hypertension, previous diabetes, Apgar score, birth order, maternal age, gestational age, birth weight, gravidity, and parity.

## Discussion

 In this study, a retrospective data analysis was used to demonstrate changes in pregnancy-related complications before and during the COVID-19 pandemic. Some indirect changes in pregnancy outcomes seem to be inevitable during this global health emergency through the disruption in maternal and perinatal health services,^4,8,20^ as the healthcare systems even in high-income countries were not fully prepared for the COVID-19 pandemic.^2^ In the current study, the rates of preeclampsia, gestational diabetes, and CS increased during the pandemic compared to the pre-pandemic period. Similarly, in the study of Sun et al,^5^ the risk of preeclampsia was reported to be higher during the pandemic period compared to the pre-pandemic period. According to Liu et al,^21^ women exposed to the initial phase of the COVID-19 pandemic in the first trimester were more likely to be diagnosed with pregnancy-related hypertension. While the exact causes for the increased occurrence of preeclampsia during the COVID-19 pandemic have not been completely elucidated, factors such as mental health disorders and lifestyle modifications may contribute to these findings. In addition, psychological risks could potentially weaken the immune system, resulting in the development of pregnancy-related hypertension. Conversely, in a single hospital-based study from Japan, a reduction was reported in pregnancy-related hypertensive disorder during the COVID-19 pandemic compared to previous years. Staying home for COVID-19 prevention, leading to an increase in women resting at home, may explain the results.^22^ A systematic review and meta-analysis also showed that there was no increased risk of the occurrence of preeclampsia among pregnant women with the COVID-19 infection, and it seems that there is no clinically significant increase in the rate of preeclampsia in the pandemic period.^23^

 A higher rate of CS was observed in the COVID-19 pandemic period than in the pre-pandemic period. This observation aligns with a comparative cross-sectional study conducted in Ethiopia, which also reported an increased rate of CS without any improvement in perinatal outcomes during the pandemic compared to the pre-pandemic period.^11^ In contrast, Einarsdóttir et al^24^ reported a decline in elective CS during the COVID-19 lockdown. This observation could potentially be attributed to the cancellation of all non-urgent surgeries during the lockdown. The increased COVID-19-related workload among healthcare staff may have raised the threshold for patient admission, leading to a decrease in elective CS procedures. Moreover, according to a systematic review, cesarean delivery increased in COVID-19-infected women.^25^ However, it should be noted that while there may be instances of serious outcomes such as intensive care unit admission or maternal death, the majority of women experience a mild clinical course of COVID-19 during pregnancy, and the infection does not have a significant effect on pregnancy outcomes. Although there have been reports of high rates of cesarean delivery in relation to COVID-19, there is currently no clinical evidence to support this mode of delivery. In fact, for the majority of cases, COVID-19 poses a significant threat to the health of the mother, and the vertical transmission of the virus has not been confirmed consistently.^26^ Based on recent evidence and the already high rate of CS in Iran,^27^ it is important not to consider COVID-19 as the sole indication for cesarean delivery.

 Furthermore, an increase was observed in gestational diabetes in the pandemic period compared to the pre-pandemic period. A case-control study also showed a notable increase in the prevalence of gestational diabetes during the COVID-19 pandemic compared to the previous year.^28^ Recent evidence has indicated that COVID-19 may potentially lead to the development of new-onset diabetes mellitus. This can occur through alterations in glucose metabolism, exacerbating the pathophysiology of preexisting diabetes, or by triggering new mechanisms altogether.^29^ In addition, the management of gestational diabetes appears to be compromised during the pandemic. This can be attributed to reduced levels of physical activity, unhealthy dietary practices, and heightened anxiety levels experienced by pregnant women.^30^ It is also probable that exposure to pandemic-related stress prior to gestational diabetes screenings causes chronic inflammation, leading to a higher risk of gestational diabetes.^28^ Therefore, there is a pressing demand for further research to comprehensively unravel the intricate mechanisms underlying the relationship between COVID-19 and gestational diabetes.^29^ Due to the heightened threat of viral transmission in the maternity units during the COVID-19 pandemic, the “one-step” approach is favored for the diagnosis of gestational diabetes. Nonetheless, overlooking the oral glucose tolerance test may result in adverse maternal, fetal, and neonatal outcomes, including preeclampsia, macrosomia, primary cesarean delivery, neonatal hypoglycemia, premature delivery, and birth injury. As a result, it is crucial to closely monitor changes in adverse outcomes in prospective studies.^31^

 To the best of our knowledge, this is the most extensive study to assess the impact of the COVID-19 pandemic on adverse pregnancy outcomes in Iran, which included over 100 000 pregnant women using a country-wide database. Although this study had a large sample size and great statistical power, which could ensure generalizability and minimize the effects of selection bias due to non-response, we were limited in using the variables included in the registers and missed information on some confounders that could lead to residual confounding. In addition, unimportant differences and shallow values close to zero possibly became statistically significant due to a large sample size effect. Further, this study focused on understanding the indirect impact of COVID-19 on adverse pregnancy outcomes, and the direct effects of COVID-19 on pregnancy were not evaluated and await future large studies. Moreover, it should be noted that the exact number of women within the database who contracted COVID-19 was unknown. Therefore, this variable should be adequately controlled in future studies. The findings of our study should be cautiously interpreted, recognizing that the *P* value is a continuous measure of evidence, and the decision to consider it significant or not is often arbitrary. For example, we encourage future studies with larger sample sizes to provide a more definitive assessment of the anemia status across the two time points.

 Finally, in this study, confounders were selected based on subject matter knowledge, the availability of indicators, and a comprehensive literature review. While these criteria guided our confounder selection process, it is important to acknowledge that we did not employ directed acyclic graphs (DAGs) to identify potential unmeasured confounders systematically. Future research in this field may benefit from incorporating DAG analysis to provide a more rigorous and comprehensive assessment of confounding variables. DAGs offer a valuable approach to visualizing causal relationships and identifying potential confounders, enhancing the robustness of causal inference in observational studies. Therefore, we recommend that future similar studies consider the use of DAGs as a powerful tool for confounder selection, in addition to subject matter knowledge, literature review, and control to further strengthen the validity of their findings.

## Conclusion

 In this extensive study, a significant association was observed between the COVID-19 pandemic and an increase in preeclampsia, gestational diabetes, and CS deliveries. These findings strongly underscore the need to prioritize ongoing monitoring of perinatal outcomes in response to the pandemic, aiming to safeguard the health of mothers and their babies, especially in light of emerging new variants of coronavirus over time. Health service delivery during the epidemic is required to ensure the early detection and timely intervention of high-risk pregnant women, and monitoring the risk of adverse perinatal outcomes is strongly recommended in follow-up visits. Further research is warranted to gain a richer understanding of the intricate interplay between the COVID-19 pandemic and pregnancy complications, especially considering the effects of new coronavirus variants. Given that the current study primarily focused on investigating the immediate effects of the pandemic on expectant mothers, it is crucial to address the potential long-term consequences through future prospective cohort studies.
